# Association of *Blastocystis* subtype 3 with elevated IL-8–mediated inflammation in patients with irritable bowel syndrome

**DOI:** 10.1186/s13099-026-00887-5

**Published:** 2026-10-21

**Authors:** Salwa M. Morsy, Magda S. A. Abdeltawab, Amal M. Mahfoz, Mahmoud A. Elkerdawy, Hanaa S. Sallam, Amira R. Ismail

**Affiliations:** 1 Department of Medical Parasitology, Faculty of Medicine, Cairo University, Cairo, Egypt; 2 Department of Medical Parasitology, Faculty of Medicine, Modern University for Technology and Information, Cairo, Egypt; 3 Department of Pharmacology and Toxicology, Egyptian Drug Authority (EDA), Giza, Egypt; 4 Hepatology, Gastroenterology and Infectious Diseases Department, Faculty of Medicine, Kafrelsheikh University, Kafrelsheikh, Egypt; 5 Department of Human Physiology, Faculty of Medicine, Suez Canal University, Ismailia, Egypt; 6 Department of Population Health and Health Disparities, Division of Global Partnerships, School of Public and Population Health, University of Texas Medical Branch at Galveston, Galveston, TX USA

**Keywords:** *Blastocystis* subtypes, Irritable bowel syndrome (IBS), Interleukin-8 (IL-8), Intestinal low-grade inflammation, Subtype-specific pathogenicity

## Abstract

**Background:**

The role of *Blastocystis* in the pathophysiology of irritable bowel syndrome (IBS) remains controversial, with emerging evidence suggesting subtype-specific differences in pathogenicity. Interleukin-8 (IL-8), a key pro-inflammatory chemokine, has been implicated in mucosal immune activation in both IBS and parasitic infections.

**Objective:**

This study aimed to investigate the association between *Blastocystis* subtypes and clinical manifestations of IBS, with a particular focus IL-8 as part of the systemic pro-inflammatory immune response associated with functional GIT disorders and intestinal infections.

**Methods:**

In this cross-sectional study, 298 symptomatic patients attending a tertiary gastroenterology clinic were evaluated using Rome IV criteria for IBS diagnosis. Identification of *Blastocystis* in stool samples was done by direct microscopic examination. Subtype identification using PCR and subtype-specific sequence-tagged site primers were also performed. A balanced random selection was used to compare serum IL-8 levels between *Blastocystis*-positive IBS patients and an equal number of randomly selected *Blastocystis*-negative IBS patients. Clinical, biochemical, and demographic variables were analyzed in relation to infection status and subtype distribution.

**Results:**

IBS was diagnosed in 197 out of 298 participants (66.1%), among whom 57 (28.9%) were positive for *Blastocystis*. Subtype analysis revealed a predominance of subtype (ST) 3 (86%), followed by ST1 (14%). Serum IL-8 levels were significantly higher in *Blastocystis*-infected IBS patients compared to non-infected controls (129.24 ± 56.28 vs. 26.28 ± 9.28 pg/mL, *P* < 0.001). Multivariate analysis identified younger age, diarrhea, and spring presentation as independent predictors of *Blastocystis* infection.

**Conclusions:**

IBS is frequently associated with *Blastocystis* infection, particularly with subtype ST3. Serum levels of IL-8 are higher in *Blastocystis*-infected IBS patients as compared to uninfected ones. Statistical comparison between IL-8 levels in both subtype groups revealed no significant difference, despite the higher numerical values among the ST3 group. The potential aggravating effect of blastocystosis on IBS symptomatology and the relation between individual subtypes and the pro-inflammatory cytokine response require further mechanistic and longitudinal studies to clarify the causal relationship between *Blastocystis* subtypes and IBS pathogenesis. In addition, local inflammatory markers should be used to study local immune modulation and identify molecular effectors implicated in the pathogenesis of *Blastocystis* in IBS -infected patients.

## Background

Irritable bowel syndrome (IBS) is a common chronic functional gastrointestinal (GI) disorder of global prevalence that serves as a primary driver of clinical consultations in gastroenterology practices worldwide [1]. Despite its recognized multifactorial etiology, the pathophysiology of IBS remains incompletely understood. Factors implicated in the pathogenesis of IBS include disruptions in gut-brain signaling, intestinal microbiota, mucosal immune homeostasis, environmental stressors, visceral hypersensitivity, altered intestinal permeability, and low-grade mucosal inflammation [2]. The diagnosis of IBS remains entirely symptom-based due to the lack of well-studied diagnostic biochemical or metabolic markers. It therefore relies on the standardized Rome IV diagnostic criteria, which define the syndrome by the presentation of recurrent abdominal pain coupled with distinct alterations in bowel habits, particularly diarrhea and constipation [3, 4].

Post-infectious IBS (PI-IBS) is a well-recognized form of the disease, which refers to the persistent onset of functional GI symptoms following an acute episode of gastroenteritis triggered by viral, bacterial, or parasitic pathogens [5]. Among potential parasitic contributors, *Blastocystis* has emerged as a non-motile anaerobic protozoon of intense scientific interest. It is, taxonomically classified within the diverse phylum Stramenopiles, and is among the most frequently identified organisms in human fecal surveys globally and displays. *Blastocystis* also displays an extraordinarily wide range of genetic diversity [6]. Although long considered as harmless commensals of the human gut, a growing body of epidemiological and experimental evidence suggests that *Blastocystis* may actively participate in structural and functional intestinal pathology, particularly within functional spectrum disorders like IBS [7].

The organism is transmitted primarily via the fecal-oral route and has a complex life cycle characterized by multiple distinct morphological phases, including vacuolar, granular, amoeboid, and cystic forms [8]. The clinical presentations often attributed to symptomatic *Blastocystis* colonization resemble cardinal IBS symptoms, such as chronic abdominal pain, diarrhea, constipation, flatulence, and systemic fatigue [9]. Regional epidemiological investigations have identified a *Blastocystis* prevalence of up to 30% to 40% among patients diagnosed with IBS across parts of Europe and the Middle East [10]. Still, a clear pathogenic role for Blastocystis cannot be conclusively defined, largely discrepancies in reported prevalence rates across target populations and variations in diagnostic methodologies.

This clinical inconsistency may arise from the extensive genetic heterogeneity found within the genus *Blastocystis*. To date, a multitude of distinct zoonotic and anthroponotic subtypes (ST1 through ST17 and beyond) have been identified across various avian, mammalian, and human hosts, with their relative distributions showing distinct geographical variations [11]. Emerging data suggest that these subtype-specific genomic backgrounds directly modulate host-parasite interactions by dictating cellular virulence potential, triggering specific host immune pathways, and reshaping the surrounding gut microbiota niche [12–14]. Consequently, evaluating *Blastocystis* at the subtype level may elucidate its clinical relevance in functional bowel disorders [15]. However, the data on the precise pathways through which specific subtypes interact with the intestinal microenvironment to trigger symptoms remain insufficient [16].

At the immunological level, *Blastocystis* colonization has been linked to the induction of localized pro-inflammatory cascades, characterized by the upregulation of cytokines that drive mucosal inflammation. In vivo and in vitro models have demonstrated that the parasite promotes cell death, compromises epithelial barrier function, and elevates oxidative stress markers along with pro-inflammatory mediators in host tissues [17]. Among these immune mediators, interleukin-8 (IL-8) plays a fundamental role as a powerful neutrophil chemoattractant and an upstream regulator of acute inflammatory pathways [18, 19]. Elevated levels of IL-8 have been reported in both inflammatory bowel diseases (IBD) and IBS and may serve as a surrogate marker of low-grade intestinal inflammation [20]. Notably, enhanced IL-8 expression has been captured in IBS cohorts infected with *Blastocystis*, signaling a possible functional link between parasitic carriage and mucosal immune activation [21].

Given this substantial genomic diversity and the varied immunomodulatory effects observed across strains, targeted work is needed to investigate the impact of specific *Blastocystis* subtypes on GIT inflammation associated with IBS. Accordingly, the present study aimed to explore the potential association between *Blastocystis* Subtype 3 (ST3) infection and elevated serum IL-8 levels in patients with irritable bowel syndrome (IBS). We also sought to analyze overall prevalence of *Blastocystis* and specific subtype distribution among IBS patients compared to controls, as well as to compare serum IL-8 levels across *Blastocystis*-positive vs. *Blastocystis*-negative subgroups. Additional targets included the exploration of the possible correlations between serum IL-8 concentration, specific clinical symptoms, and demographic characteristics.

## Materials and methods

### Ethical considerations

The study was conducted in accordance with the ethical principles of the Declaration of Helsinki (2013) and received approval from the Ethical Committee of the Faculty of Medicine, Kafr El-Sheikh University (Approval No. KFSIRB200-921).

### Study design and population

This cross-sectional study included 298 symptomatic patients recruited from the gastroenterology outpatient clinic at Kafr El-Sheikh University Hospital. Inclusion criteria comprised patients presenting with gastrointestinal symptoms, including diarrhea, vomiting, abdominal pain, and flatulence. IBS diagnosis was done by an experienced consultant gastroenterologist based on the Rome IV diagnostic criteria through a detailed clinical assessment. The diagnosis was established after a comprehensive medical history and physical examination, with exclusion of organic gastrointestinal diseases through appropriate clinical evaluation and relevant investigations when clinically indicated. Other exclusion criteria included recent use of antimicrobial or antiparasitic agents, presence of other parasitic infections detected by stool examination, immunocompromised status, and the presence of alarming signs such as history of gastrointestinal bleeding or unexplained weight loss. Patients diagnosed with celiac disease or thyroid disorders were also excluded.

Based on clinical and parasitological findings, participants were categorized into four groups:

- Group IA: IBS patients infected with *Blastocystis*.
- Group IB: IBS patients free from *Blastocystis* infection.
- Group IIA: Non-IBS patients infected with *Blastocystis*.
- Group IIB: Non-IBS patients free from *Blastocystis* infection.

Patients with confirmed *Blastocystis* infection were further classified according to the predominant subtype identified by molecular analysis.

### Sample size calculation

The sample size was calculated using the standard formula for cross-sectional studies based on an expected prevalence of irritable bowel syndrome of 13% among Egyptian adults, as previously reported by El Sharawy et al. (2022) [22]. Assuming 95% confidence level with margin of error 5% so the minimum required sample size estimated to be 174 participants. The final study population included 298 participants to ensure adequate representation of *Blastocystis*-positive cases in a real-world outpatient setting. Since sampling was conducted prior to subtyping, the subtypes detected and their prevalence among the study participants could not be included as determining factors in the initial sample size calculation. Given the higher number of non-infected IBS patients compared to the *Blastocystis*-infected cohort, an equal number of non-infected patients was selected via blind, simple random sampling to establish balanced groups for the comparison between IL-8 levels in *Blastocystis*-positive and *Blastocystis*-negative IBS patients.

### Sample collection

Morning stool samples were collected for parasitological examination. A portion of each sample was fixed in formalin for concentration techniques, while another portion was stored frozen at -20 °C for molecular analysis. Blood samples were collected, and serum was separated and stored at -20 °C for IL-8 quantification.

Participants who provided stool samples on three alternate days were included. Written informed consent was obtained from all participants, and data confidentiality was ensured through anonymization.

### Parasitological examination

#### Stool examination

Stool samples underwent macroscopic examination for the presence of mucus, blood, or helminths. Microscopic examination was performed for all three samples collected from each patient using unstained and iodine-stained wet mounts to identify *Blastocystis* and exclude the presence of other parasites. Samples negative on direct examination were re-evaluated following formalin–ethyl acetate sedimentation concentration. Identification of *Blastocystis* was done by a consultant parasitologist based on the morphological criteria of Blastocystis forms (vacuolar, granular, amoeboid, and cyst forms). Parasitological examination for *Blastocystis* was done as a selection procedure for samples to be subjected for molecular identification and genotyping. Samples that were positive for *Blastocystis* by parasitological examination but negative by molecular identification were excluded from further analysis [23].

#### Detection of stool reducing sugars

Reducing sugars were assessed using Benedict’s test. Fresh stool samples were diluted with distilled water at a ratio of 1:2, followed by the addition of 2 mL Benedict’s reagent. The mixture was heated in a boiling water bath for 3–5 min, and color change was used to indicate the presence of reducing sugars. A positive control (1% glucose solution) and a negative control (distilled water) were included in each daily testing batch to assure quality control of the test [24].

#### Determination of stool pH

Stool pH was measured using commercial reagent strips (Medi-Test Combi 10R SGL, LOT no. 67111). A small sample was applied to the strip, and results were read after 30 s according to the manufacturer’s reference scale. The strips, containing methyl red and bromothymol blue, detect pH values ranging from 5.0 to 9.0.

Before performing daily testing, batch performance and indicator integrity were validated using standard reference buffer solutions (pH 4.0, 7.0, and 10.0). All test strips were stored and handled strictly in accordance with manufacturer guidelines to prevent ambient humidity degradation.

### Molecular Identification and Genotyping of *Blastocystis*

To establish the presence and specific subtype distribution of *Blastocystis*, a structured, two-phase molecular approach was deployed. Total genomic DNA (gDNA) was isolated from all collected fecal specimens using the QIAamp^®^ DNA Mini Kit (Qiagen, Hilden, Germany), strictly adhering to the manufacturer’s customized stool-processing protocols. The extracted gDNA served as the template for both subsequent PCR phases. Every primary universal PCR and subtyping amplification run systematically included a negative control (nuclease-free water substituted for template DNA) to monitor for aerosol contamination, alongside a positive control consisting of verified *Blastocystis* DNA. Ambiguous samples were subjected to repeat DNA extraction from original stool aliquots and re-amplified in duplicate.

#### Phase 1: Universal Screening and Detection

In the first phase, all 298 stool samples included in the study population were screened using universal diagnostic primers targeting the small subunit ribosomal RNA (SSU rDNA) gene, following standard diagnostic protocols [25]. This universal PCR assay utilized the following primer pairs:

- **RD5 (Forward)**: 5’-GGAGGTAGTGACAATATC-3’.
- **BhRDr (Reverse)**: 5’-TGTTATCCTCACTAACTTCG-3’.

This assay was designed to amplify a diagnostic fragment of approximately 600 bp to confirm *Blastocystis*-positive status.

The screening PCR reactions were carried out in a 25 µL total volume containing:

- 12.5 µL of 2X PCR Master Mix.
- 1.0 µL of RD5 primer (10 pmol/µL).
- 1.0 µL of BhRDr primer (10 pmol/µL).
- 0.1 µL of Taq DNA Polymerase.
- 7.4 µL of nuclease-free distilled water.
- 3.0 µL of purified gDNA template.

Amplification was driven in a thermal cycler under the following specific screening conditions: an initial denaturation plateau at 94 ^∘^C for 4 min; followed by 35 continuous cycles of denaturation at 94 ^∘^C for 60 s, primer annealing at 55 ^∘^C for 60 s, and extension at 72^∘^C for 80 s; culminating in a final elongation step at 72 ^∘^C for 5 min [26].

Amplicons were resolved via horizontal electrophoresis on a 1.5% agarose gel, stained with ethidium bromide, and visualized under ultraviolet transillumination. Out of the 298 total samples screened, this phase yielded exactly 57 laboratory-confirmed positive results (all within the IBS-positive cohort), which were then moved forward into the next phase.

#### Phase 2: Sequence-Tagged Site (STS) Subtype Genotyping

In the second phase, specific subtype characterization was performed exclusively on the 57 *Blastocystis*-positive samples identified during Phase 1. Genotyping was executed via multiplexed PCR using seven distinct, standardized sequence-tagged site (STS) primer sets (ST1 through ST7, Table 1), running each diagnostic primer set in a separate, independent reaction tube to avoid cross-reactivity, according to the established classification system [26].

The subtyping PCR reaction mixtures (25 µL final volume) consisted of:

- 12.5 µL of 2X PCR Master Mix.
- 1.0 µL of the respective specific STS Forward primer (10 pmol/µL).
- 1.0 µL of the respective specific STS Reverse primer (10 pmol/µL).
- 0.1 µL of Taq Polymerase.
- 8.4 µL of nuclease-free distilled water.
- 2.0 µL of the template SSU rDNA amplicon generated in Phase 1.

The specific thermal cycling sequence for this downstream STS amplification was programmed as follows: an initial denaturation step at 94 °C for 4 min; followed by 40 repetitive cycles of denaturation at 94 °C for 30 s, primer annealing at 58 °C for 30 s, and extension at 72 °C for 60 s; with a final extension plateau at 72 °C for 5 min [26].

The resulting subtype-specific amplicons were separated via electrophoresis on a 1.5% agarose gel, stained, and evaluated against expected molecular weight indices to sort the 57 positive isolates into their respective genomic subtypes (yielding 49 ST3 isolates and 8 ST1 isolates).

**Table 1:** Sequence of standardized subtype-specific Sequence –Tagged-Site (STS) forward (F) and reverse (R) primers and their expected PCR product size

Sub-type	STS Primers		Sequence	Expected product size (bp)
1	SB83	F	5’- GAAGGACTCTCTGACGGTGA-3’	351 bp
		R	5’-GTCCAAATGAAAGGCAGC-3’	
2	SB155	F	5’-ATCAGCCTACAATCTCCTC-3’	650 bp
		R	5’-ATCGCCACTTCTCCAAT-3’	
3	SB227	F	5’-TAGGATTTGGTGTTTGGAGA-3’	526 bp
		R	5’-TTAGAAGTGAAGGAGATGGAAG-3’	
4	SB332	F	5’-GCATCCAGACTACTATCAACATT-3’	338 bp
		R	5’-CCATTTTCAGACAACCACTTA-3’	
5	SB340	F	5’-TGTTCTTGTGTCTTCTCAGCTC-3’	704 bp
		R	5’-TTCTTTCACACTCCCGTCAT-3’	
6	SB336	F	5’-GTGGGTAGAGGAAGGAAAACA-3’	317 bp
		R	5’-AGAACAAGTCGATGAAGTGAGAT-3’	
7	SB337	F	5’-GTCTTTCCCTGTCTATTCTGCA-3’	487 bp
		R	5’-AATTCGGTCTGCTTCTTCTG-3’	

### Measurement of IL-8 by ELISA

Serum IL-8 levels were measured in *Blastocystis*-positive IBS patients and an equal number of randomly selected *Blastocystis*-negative IBS controls. Blood samples were collected in standard tubes, allowed to clot, and centrifuged at 2,000 × g for 10 min. Serum was separated and stored at -20 °C until analysis.

Quantification was performed using a commercial sandwich enzyme-linked immunosorbent assay (Human IL-8 SimpleStep ELISA^®^ Kit, Cat. No. ab214030, Abcam, Cambridge, UK). All test serum samples, calibration standards (3.91–250.00 pg/mL), and blank controls were assayed in duplicate. Briefly, 12.5 µL of each sample or standard and 12.5 µL of antibody cocktail were added to antibody-coated wells and incubated for 1 h at room temperature on a plate shaker (700 rpm). Wells were washed three times with 1X Wash Buffer PT, followed by the addition of 25 µL TMB development solution and incubation in the dark on a plate shaker. The reaction was stopped before signal saturation using 25 µL of the stop solution, and optical density was measured at 450 nm using a microplate reader.

The linear dynamic detection range of the assay was 3.91–250.00 pg/mL with an analytical sensitivity (minimal detectable dose) of 1.8 pg/mL. Standard curves demonstrated high linearity across all plates (R² > 0.99). Assay reproducibility was confirmed with intra-assay and inter-assay coefficients of variation (CV) of 1.8% and 7.5%, respectively, with all sample readings falling within the validated linear dynamic range.

### Statistical analysis

The primary outcome was to explore the potential association between *Blastocystis* Subtype 3 (ST3) infection and elevated serum IL-8 levels in patients with irritable bowel syndrome (IBS). The secondary outcome was to analyze overall prevalence and subtype distribution of *Blastocystis* among IBS patients compared to controls, as well as general comparisons of serum IL-8 levels across *Blastocystis*-positive vs. *Blastocystis*-negative subgroups. Additional outcomes include the exploration of the possible correlations between serum IL-8 concentration, specific clinical symptoms, and demographic characteristics.

Data was coded and analyzed using the Statistical Package for the Social Sciences (SPSS) version 28 (IBM Corp., Armonk, NY, USA). Quantitative variables were summarized using mean ± standard deviation (SD), median, minimum, and maximum values, whereas categorical variables were expressed as frequencies and percentages.

Comparisons between quantitative variables were performed using the non-parametric Mann–Whitney U test [27]. Comparisons between categorical variables were conducted using the chi-square (χ²) test, and Fisher’s exact test was applied when the expected cell frequency was less than 5 [28]. For the comparison between serum IL-8 level in *Blastocystis*-positive versus negative IBS patients, balanced, random sampling was applied, where *Blastocystis*-infected IBS patients (*N* = 57) were compared to an equal number of uninfected ones. Multivariate binary logistic regression analysis was performed to identify independent predictors of *Blastocystis* infection. Parameters showing statistical significance (*P* < 0.05) during the univariable analysis were further analyzed by multivariate binary logistic regression. Results were expressed as odds ratios (ORs) with 95% confidence intervals (CIs) [29]. Statistical significance was defined at P-value < 0.05.

## Results

### Characteristics of the study population

In the current study, 298 patients attending the gastroenterology outpatient clinic were evaluated for the presence of IBS. The clinical diagnosis of IBS was confirmed in 197 patients (66.1%). Patients’ personal data are represented in Table 2.

**Table 2:** Personal data of the study population

Personal data		IBS				
		Positive		Negative		P value
		Count	%	Count	%	
Age group	< 20	43	21.8%	23	22.8%	0.501
	20–29	51	25.9%	28	27.7%	
	30–39	51	25.9%	27	26.7%	
	40–49	31	15.7%	8	7.9%	
	50–59	11	5.6%	7	6.9%	
	> 60	10	5.1%	8	7.9%	
Gender	Male	110	55.8%	59	58.4%	0.671
	Female	87	44.2%	42	41.6%	

### Clinical presentation among the study participants

Patients presented to the gastroenterology clinic with a variety of symptoms such as diarrhea, vomiting, abdominal pain, and flatulence. Diarrhea was more frequently reported among IBS patients (Table 3).

**Table 3:** GIT symptoms among the study population

GIT symptoms		IBS				
		Positive		Negative		*P* value
		Count	%	Count	%	
Diarrhea	Present	165	83.8%	73	72.3%	0.019^*^
	Absent	32	16.2%	28	27.7%	
Abdominal pain	Present	187	94.9%	92	91.1%	0.200
	Absent	10	5.1%	9	8.9%	
Flatulence	Present	137	69.5%	72	71.3%	0.756
	Absent	60	30.5%	29	28.7%	
Vomiting	Present	31	15.7%	16	15.8%	0.981
	Absent	166	84.3%	85	84.2%	
Seasonal variation of symptomatology	Spring	53	26.9%	16	15.8%	0.006*
	Summer	66	33.5%	27	26.7%	
	Autumn	52	26.4%	30	29.7%	
	Winter	26	13.2%	28	27.7%	

Fischer’s exact test; *Statistical significance at *P* < 0.05

### Macroscopic and biochemical stool characteristics in relation to the diagnosis of IBS

Macroscopic examination of stool samples from IBS patients showed blood in stool in 51 samples (25.9%) as compared to 28 samples (27.7%) in the IBS negative group (*P* = 0.734). Mucus was present in 171 samples (86.8%) from IBS patients, and in 90 samples (89.1%) from IBS negative patients (0.568). In this cohort, all IBS-positive samples demonstrated an acidic reaction and the presence of reducing sugar, whereas all IBS negative samples were alkaline and were devoid of reducing sugar (*P* < 0.001).

### Molecular identification and characterization of *Blastocystis*

*Blastocystis* were detected by PCR in 57 patients diagnosed with IBS (28.9%). In contrast, all samples from IBS-free patients were negative for *Blastocystis* (*P* < 0.001).

### Study parameters in relation to the presence of *Blastocystis*

**Fig. 1:**
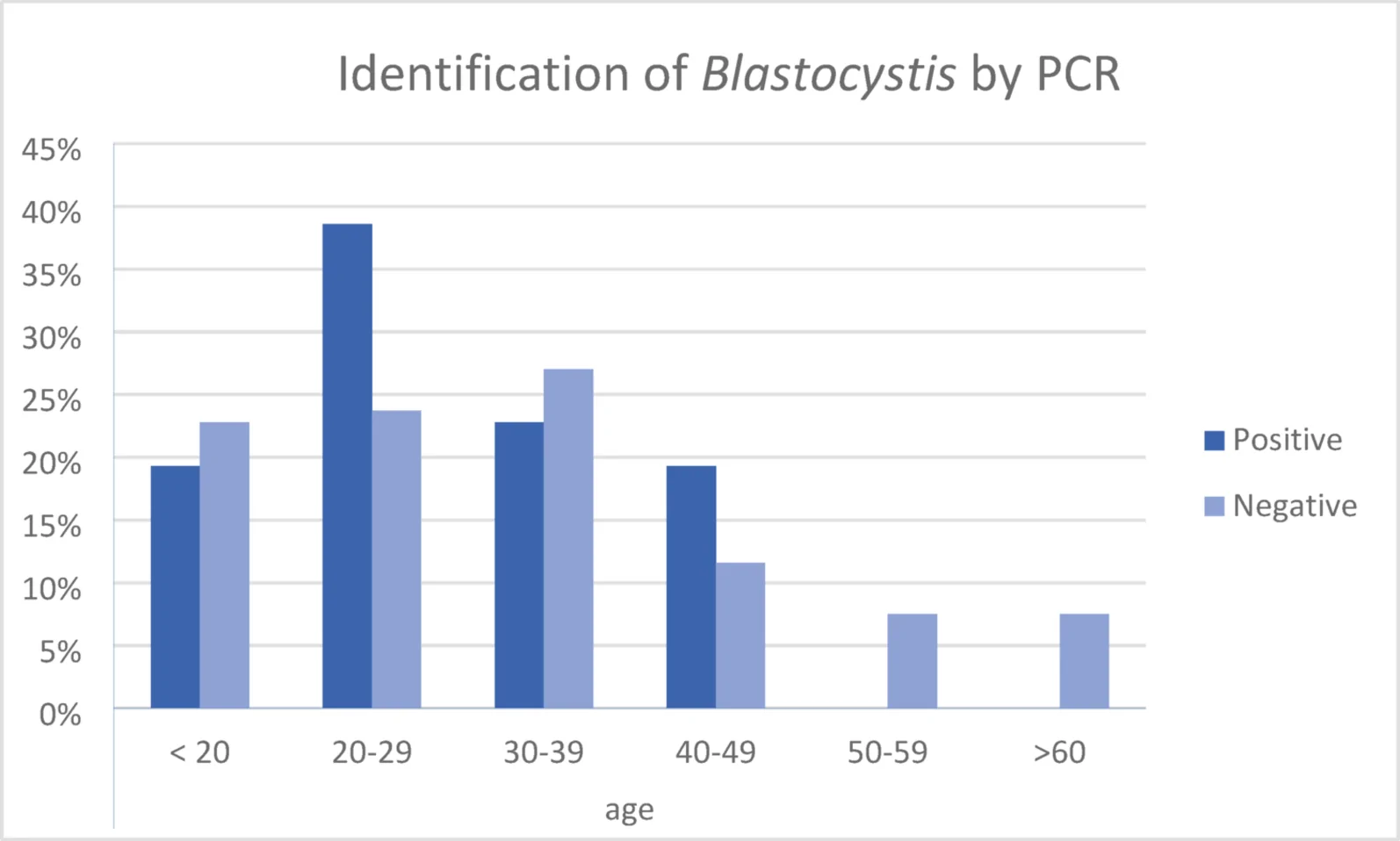
Bar chart showing the percentage of patients infected with *Blastocystis* as confirmed by PCR in relation to their age groups

Patients diagnosed with IBS and infected with *Blastocystis* included 34 male patients (59.6%) and 23 female patients (40.4%). In comparison, *Blastocystis*-free IBS patients included 76 males (54.3%) and 64 females (45.7%). The highest prevalence of *Blastocystis* infection was found among the 20 to 29-years age group (38.6%). Infection was absent in patients above 50 years of age (Fig. 1).

### GIT symptoms in *Blastocystis*-infected versus non-infected IBS patients

Diarrhea, abdominal pain, and vomiting were significantly more prevalent among infected patients. Symptoms were more frequently reported during spring and summer as compared to autumn and winter (Table 4).

**Table 4:** Comparison between the prevalence of symptoms and seasonal variation in clinical presentation in *Blastocystis*-infected and uninfected IBS patients

		**PCR**				
		**Positive**		**Negative**		***P*** **value**
		**Count**	**%**	**Count**	**%**	
Diarrhea	Present	53	93.0%	185	76.8%	0.006*
	Absent	4	7.0%	56	23.2%	
Abdominal pain	Present	57	100.0%	222	92.1%	0.030*
	Absent	0	0.0%	19	7.9%	
Flatulence	Present	40	70.2%	169	70.1%	0.994
	Absent	17	29.8%	72	29.9%	
Vomiting	Present	15	26.3%	32	13.3%	0.015*
	Absent	42	73.7%	209	86.7%	
Season	Spring	28	49.1%	41	17.0%	< 0.001*
	Summer	19	33.3%	74	30.7%	
	Autumn	6	10.5%	76	31.5%	
	Winter	4	7.0%	50	20.7%	

Fischer’s exact test; *Statistical significance at *P* < 0.05

### Macroscopic and biochemical stool characteristics in relation to the molecular diagnosis of *Blastocystis* infection

**Fig. 2:**
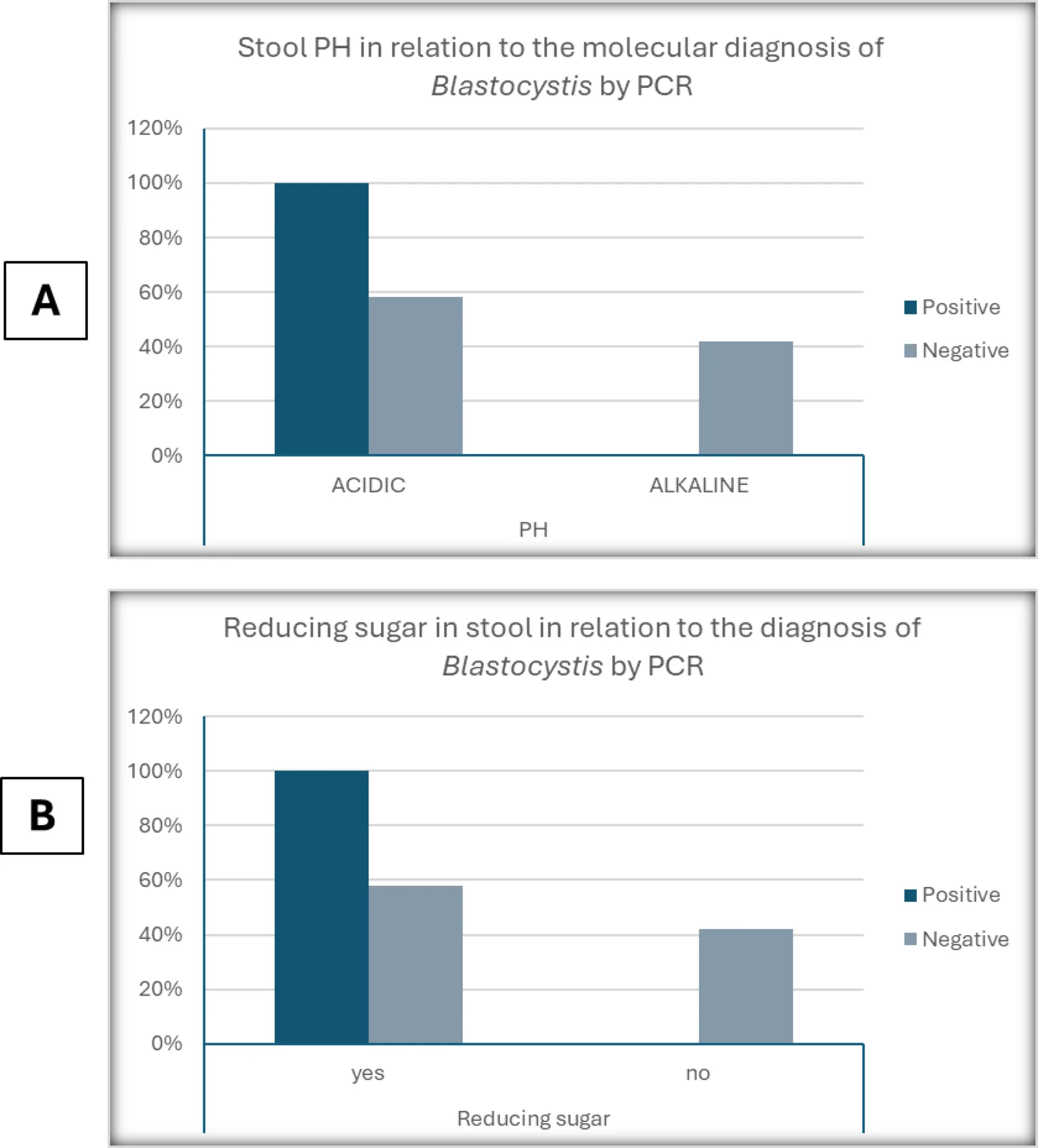
Bar charts showing PH (**A**) and reducing sugar in stool (**B**) in *Blastocystis* negative and positive samples

Blood was detected by macroscopic examination in 17 *Blastocystis* positive samples (29.8%) and in 62 *Blastocystis* negative samples (27.5%) (*P* = 0.528). Mucus was present in 47 cases infected with *Blastocystis* (82.5%), and in 214 cases free of the infection (88.8%) (*P* = 0.192). As regards the chemical reactions of the examined stool samples, all *Blastocystis* positive samples were acidic and positive for the presence of reducing sugar, whereas *Blastocystis* free samples were positive in only 58.1% of cases (*N* = 140) for both criteria (*P* < 0.001) (Fig. 2).

### Study parameters in relation to *Blastocystis* subtypes

*Blastocystis* subtype 3 (ST3) was more predominant among IBS patients (49 out of 57 patients; 86%), as compared to 8 patients only (14%) being infected with subtype 1 (ST1).

**Fig. 3:**
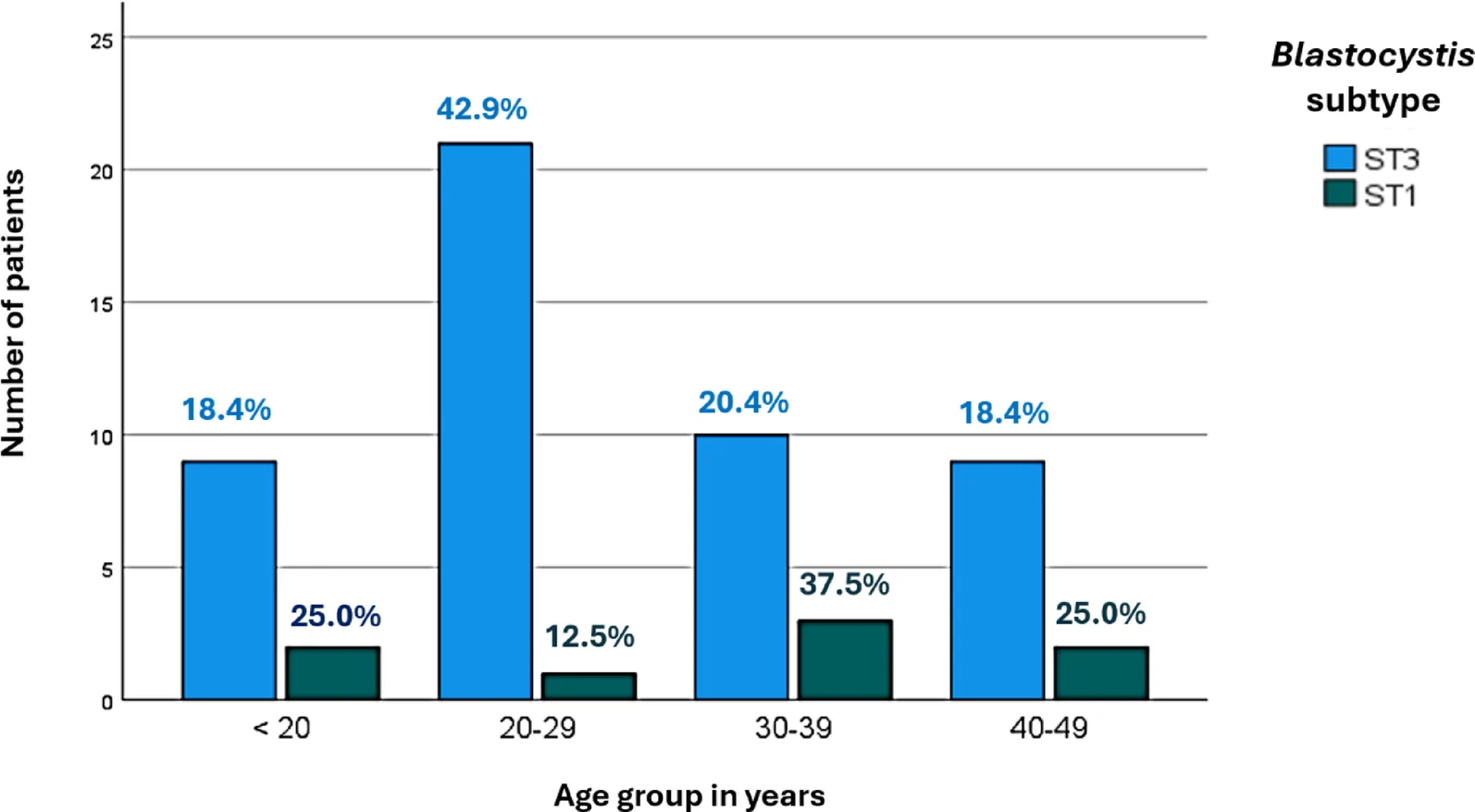
Bar chart showing number and percent of patients in the different age groups according to their Blastocystis-subtype infection status

**Fig. 4:**
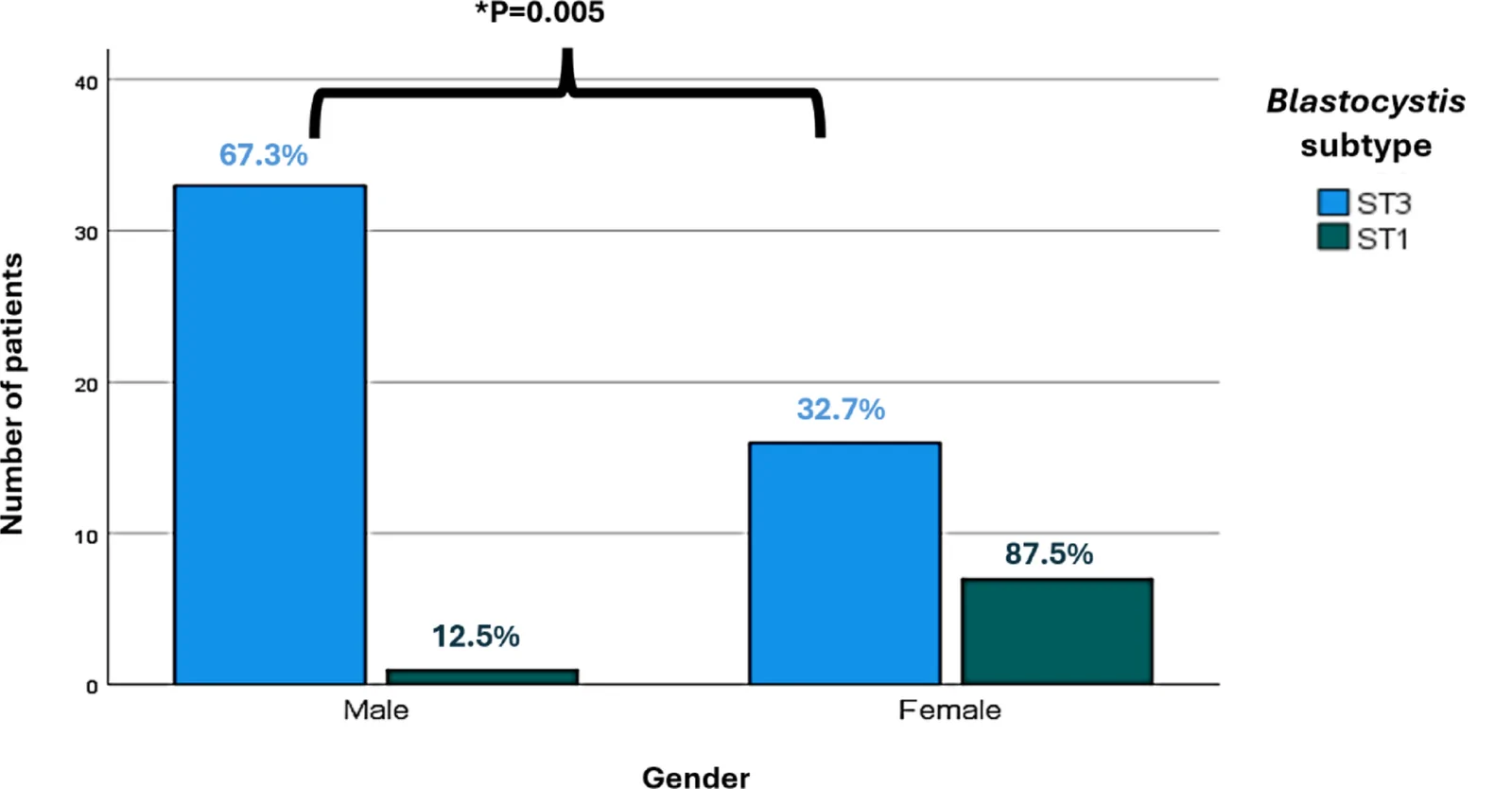
Bar chart showing number and percent of male and female patients in relation to their *Blastocystis*-subtype infection status

ST3 was more prevalent in male patients (67.3%), while ST1 was more prevalent among female patients (87.5%). Comparison of other personal and clinical data and stool analysis revealed no significant difference between the two subtype groups (Figs. 3 and 4; Tables 5 and 6).

**Table 5:** Comparison of GIT symptoms between *Blastocystis* ST3 and ST1

GIT symptoms		Blastocystis subtypes				
		ST3		ST1		*P* value
		Count	%	Count	%	
Diarrhea	Present	45	91.8%	8	100.0%	1
	Absent	4	8.2%	0	0.0%	
Abdominal pain	Present	49	100.0%	8	100.0%	-------
Flatulence	Present	34	69.4%	6	75.0%	1
	Absent	15	30.6%	2	25.0%	
Vomiting	Present	15	30.6%	0	0.0%	0.095
	Absent	34	69.4%	8	100.0%	
Season	Spring	24	49.0%	4	50.0%	1
	Summer	16	32.7%	3	37.5%	
	Autumn	5	10.2%	1	12.5%	
	Winter	4	8.2%	0	0.0%	

Fischer’s exact test; *Statistical significance at *P* < 0.05

**Table 6:** Comparison of macroscopic stool examination findings between *Blastocystis* ST3 and ST1

Macroscopic stool examination		Blastocystis subtypes				
		ST3		ST1		*P* value
		Count	%	Count	%	
Blood	Present	16	32.7%	1	12.5%	0.413
	Absent	33	67.3%	7	87.5%	
Mucous	Present	42	85.7%	5	62.5%	0.137
	Absent	7	14.3%	3	37.5%	
PH	Acidic	49	100.0%	8	100.0%	------
Reducing sugar	Present	49	100.0%	8	100.0%	-------

Fischer’s exact test; *Statistical significance at *P* < 0.05

### Serum IL-8 levels among study participants

**Fig. 5:**
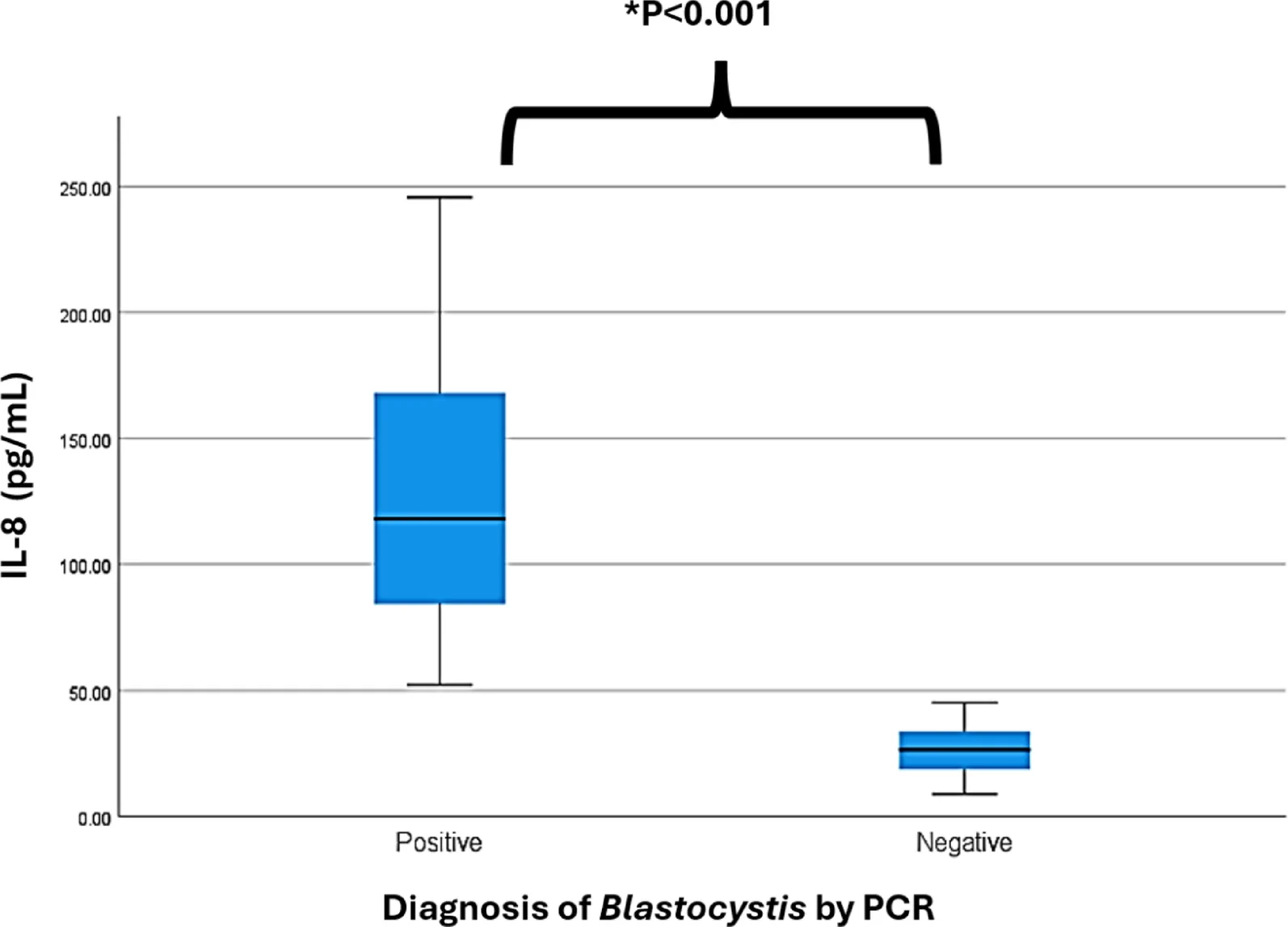
Boxplot showing comparison of medians and interquartile ranges (IQRs) of IL-8 levels (pg/mL) in *Blastocystis* positive and negative patients; *Statistical significance at *P* < 0.05

Serum IL-8 levels were assessed in *Blastocystis*-infected IBS patients and an equal number of randomly selected uninfected IBS patients. Comparison between personal and clinical data of both groups is represented in Tables 7 and 8. Diarrhea and abdominal pain were significantly more frequently reported by *Blastocystis*-infected patients as compared to uninfected ones (*P* = 0.003 and 0.006, respectively). Evaluation of IL-8 levels between these two randomly allocated groups revealed that IL-8 levels were significantly higher in *Blastocystis*-positive IBS patients (129.24 ± 56.28 pg/mL; median = 118.08 pg/mL) as compared to *Blastocystis*-negative ones (26.28 ± 9.28 pg/mL; median = 26.40 pg/mL) (*P* < 0.001) (Fig. 5).

**Table 7:** Comparison between personal data of IBS patients infected with *Blastocystis* and a balanced randomly selected cohort of uninfected IBS patients

Personal data		IBS patients				
		Infected with Blastocystis		Uninfected		P value
		Count	%	Count	%	
Age	< 20	11	19.3%	8	14.0%	0.011^*^
	20–29	22	38.6%	14	24.6%	
	30–39	13	22.8%	20	35.1%	
	40–49	11	19.3%	6	10.5%	
	50–59	0	0.0%	3	5.3%	
	> 60	0	0.0%	6	10.5%	
Gender	Male	34	59.6%	38	66.7%	0.437
	Female	23	40.4%	19	33.3%	

Fischer’s exact test; *Statistical significance at *P* < 0.05

**Table 8:** Comparison between GIT symptoms reported by IBS patients infected with *Blastocystis* and a balanced randomly selected cohort of uninfected IBS patients

GIT symptoms		IBS patients				
		Blastocystis-infected		Uninfected		P value
		Count	%	Count	%	
Diarrhea	Present	53	93.0%	41	71.9%	0.003^*^
	Absent	4	7.0%	16	28.1%	
Abdominal pain	Present	57	100.0%	49	86.0%	0.006^*^
	Absent	0	0.0%	8	14.0%	
Flatulence	Present	40	70.2%	43	75.4%	0.528
	Absent	17	29.8%	14	24.6%	
Vomiting	Present	15	26.3%	7	12.3%	0.058
	Absent	42	73.7%	50	87.7%	
Seasonal variation	Spring	28	49.1%	9	15.8%	< 0.001^*^
	Summer	19	33.3%	20	35.1%	
	Autumn	6	10.5%	24	42.1%	
	Winter	4	7.0%	4	7.0%	

Fischer’s exact test; *Statistical significance at *P* < 0.05

Comparison between mean and median levels of IL-8 in both *Blastocystis* subtype groups revealed numerically higher values in ST3 patients; however, with no statistical significance (*P* = 0.313). The absence of statistical significance may be partly attributable to the limited sample size of the ST1 subgroup (Table 9).

**Table 9:** IL-8 levels (pg/mL) in relation to *Blastocystis* subtype

-	ST3			ST1				*P value*
	Mean	SD	Median	Mean	SD	Median		0.313
IL-8 (pg/mL)	132.25	56.05	121.30	110.81	57.81	88.00		-

### Multivariate predictors of *Blastocystis* infection

Parameters showing statistical significance (*P* < 0.05) during the univariable analysis were further analyzed by multivariate binary logistic regression, which identified several independent predictors of *Blastocystis* infection. Increasing age was associated with a lower likelihood of infection (OR = 0.761, 95% CI: 0.610–0.950, *P* = 0.016), whereas diarrhea was independently associated with increased odds of infection (OR = 3.102, 95% CI: 1.020–9.432, *P* = 0.046). Seasonal variation was also significantly associated with infection (*P* < 0.001), with spring showing the strongest association (OR = 7.736, 95% CI: 2.466–24.267, *P* < 0.001) (Table 10).

**Table 10:** Multivariate binary logistic regression analysis for predictors of *Blastocystis* infection

-		***P*** **value**	**OR**	**95% C.I.**	
				**Lower**	**Upper**
*Blastocystis* positive patients	Age	0.016*	0.761	0.610	0.950
	Diarrhea	0.046*	3.102	1.020	9.432
	Season	< 0.001*			
	Spring	< 0.001*	7.736	2.466	24.267
	Summer	0.051	3.132	0.994	9.867
	Autumn	0.996	1.003	0.266	3.782

*Statistical significance at *P* < 0.05

## Discussion

Irritable bowel syndrome (IBS) is a prevalent functional gastrointestinal disorder that constitutes a substantial health burden and is characterized by chronic abdominal pain and altered bowel habits [30, 31]. In the present study, IBS was diagnosed in 66.1% of symptomatic patients, presenting a higher prevalence when compared to reports from other Egyptian and international population-based studies. In a meta-analysis conducted by Arif et al. spanning from 2006 to 2024, the global prevalence of IBS was found to be 14.1% across 52 countries [31]. El-Sharawy et al. reported an IBS prevalence of 27.5% among a cohort of 182 medical students from Egypt [22]. In another Egyptian study, Kasemy et al. reported a higher occurrence of IBS among health care workers (14.4%) as compared to controls (8.8%) [32]. Similarly, Abdel Latif et al. found a higher frequnency of IBS among teachers in Egypt (19.4%) as compared to controls (7.4%) [33]. The higher prevalence detected in our study as compared to these reports could be explained by the targeted selection of patients complaining of GIT symptoms, rather than the recruitment from the general population. Patients were classified according to Rome IV criteria, which have improved the clinical identification of IBS by analyzing the nature of GIT symptoms, taking into consideration symptom onset, frequency and duration [4]. Diarrhea, abdominal pain, and flatulence were the most frequent symptoms reported by our study cohort, with diarrhea being significantly more prevalent among IBS patients. These findings align with previous studies reporting diarrhea-predominant IBS as a common clinical subtype [30].

Biochemical analysis of stool samples revealed a predominance of acidic pH and the presence of reducing sugars among IBS patients. While these findings may reflect underlying carbohydrate malabsorption and altered fermentation processes within the gut, they should be interpreted with caution given the potential influence of dietary factors and the semi-quantitative nature of the assessment methods. Previous studies have linked acidic stool pH to intestinal inflammation and altered metabolic activity, including increased short-chain fatty acid production and osmotic diarrhea [34, 35]. Carbohydrate malabsorption has also been widely reported in IBS patients and may contribute to symptom generation [36, 37]. El Saftawy et al. observed increased fecal lactate dehydrogenase (LDH) levels in *Blastocystis* ST3 infected patients, which could reflect disturbances in the glycolytic pathway and increased mucosal injury [38]. Validation of the biochemical and metabolic associations between *Blastocystis* and its subtypes should be sought using mechanistic studies and multi-omics approaches.

The role of infectious agents in IBS, particularly in post-infectious forms of the disease, is increasingly recognized [39]. *Blastocystis* has been proposed as a potential contributor to the pathophysiology of IBS through both direct and indirect mechanisms, including the induction of mucosal inflammation and the modulation of gut microbiota [40]. In the current study, *Blastocystis* infection was detected in 28.9% of IBS patients and was absent in IBS-negative individuals. These findings are consistent with reports demonstrating a higher prevalence of *Blastocystis* among IBS patients in certain populations [41, 42]. However, conflicting evidence from studies conducted in Denmark and Iran have reported higher prevalences among control subjects [43, 44], which may be attributed to the potential influence of geographic, environmental, and microbiome-related factors on host-parasite interactions.

*Blastocystis* stands out among other parasites for its high genetic diversity. The presence of multiple subtypes may explain the variable clinical presentations associated with blastocystosis. In the current study, genotyping was performed via multiplexed PCR using seven distinct, standardized sequence-tagged site (STS) primer sets (ST1 through ST7) to cover the most frequently reported human-associated subtypes. In the present study, ST3 was the most frequently identified subtype, followed by ST1. ST3 has also been described as the most prevalent subtype in studies from Egypt and other regions [45–47]. In a previous study targeting patients complaining of GIT disturbances, ST3 was found to be the dominant infective subtype among 70.5% of patients as compared to 29.5% of patients that were infected with ST4 [11].

The relatively small number of ST1-infected patients detected in our study led to the limitation of the statistical power. Subtype-specific observations should therefore be described as exploratory. Large multicenter studies are required to achieve balanced subtype groups for sound statistical comparison. In addition, experimental studies comparing subtype-specific infection can help investigate differences in pathogenesis and immune reaction.

The effect of blastocystosis on host immune response may explain the pathogenic potential of the parasite. The activation of NF-κB and the induction of pro-inflammatory cytokines have been suggested to be key mechanisms for *Blastocystis*-induced immune modulation [19]. IL-8, a central mediator of neutrophil recruitment and mucosal inflammation, has been implicated in both IBS and parasitic infections [48, 49]. In the present study, serum IL-8 levels were significantly increased in *Blastocystis*-infected IBS patients compared with non-infected individuals, supporting the hypothesis that the parasite may contribute to systemic inflammatory activation. These findings are consistent with previous reports demonstrating increased IL-8 expression in IBS patients and in those infected with *Blastocystis* [21]. It is to be noted, however, that serum cytokine levels might as well reflect systemic immune activation, rather than IBS-induced immune response. Local inflammatory markers such mucosal biopsies are therefore more informative of IBS-specific intestinal pathology. Since IBS is associated with low-grade intestinal inflammation, patients are not commonly indicated for invasive procedures. This limits the availability of local tissue samples obtained from endoscopy and biopsy to a limited number of patients, which hinders the recruitment of a sufficient sample size to adequately investigate IBS in association with *Blastocystis* infection. Serum cytokine assessment is therefore a rather preliminary and exploratory index, from which causal relationships should not be concluded. The comparison between systemic and local intestinal cytokine response as indicators for IBS pathology has been discussed in a systematic review conducted by Bashashati et al. In this review the authors present reports on elevated serum pro-inflammatory cytokines such as TNF-α, IL-6 and IL-8 levels in IBS patients. The authors also describe discrepancies between reports on serum and intestinal cytokine levels, particularly IL-10, which did not show significant changes when detected in serum, whereas other studies report lower colonic IL-10 mRNA expression in IBS patients [50]. Caviglia et al. investigated the effect of the probiotic bacterium *Bifidobacterium Longum* ES1 on serum zonulin, Il-6, IL-8, and IL-12 in IBS patients. While reporting significant reduction in serum cytokine levels, the authors interpreted their findings with caution describing their work as a preliminary study [51].

Multivariate analysis identified younger age, diarrhea, and seasonal presentation as independent predictors of *Blastocystis* infection. These findings point to the importance of the role of environmental and host-related factors in parasite acquisition and clinical presentation. Presentation during spring showed the strongest association with infection, which may reflect the importance of seasonal variation and temperature on infection transmission. Environmental factors are important determinants that are often neglected and need to be validated by further epidemiological investigation.

Because the non-infected study population is larger than the infected cohort, we utilized a blind random selection process to establish equally sized groups for IL-8 evaluation. In order to avoid investigator selection bias, we kept the selection process entirely randomized and blind to baseline patient characteristics. Although IL-8 levels were numerically higher in patients infected with ST3 compared to ST1, this difference did not reach statistical significance, likely due to the limited sample size of the ST1 group. Nevertheless, the observed trend may suggest subtype-specific differences in inflammatory potential. This observation, however, can only be stated as exploratory, considering the imbalanced sample size between both subtype groups. Experimental evidence indicates that *Blastocystis* may induce intestinal inflammation through multiple mechanisms, including disruption of epithelial barrier integrity, degradation of the mucus layer via cysteine proteases, and stimulation of pro-inflammatory cytokine release [52]. These processes may act synergistically with the low-grade inflammation characteristic of IBS, potentially exacerbating symptom severity.

## Conclusion and limitations

IBS is frequently associated with *Blastocystis* infection, particularly with ST3. Serum levels of IL-8 are higher in *Blastocystis*-infected IBS patients as compared to uninfected ones with numerically higher trends in ST3 as compared to ST1 infected participants. The potential aggravating effect of blastocystosis on IBS symptomatology and the relation between individual subtypes and the pro-inflammatory cytokine response require further mechanistic and longitudinal studies to clarify the causal relationship between *Blastocystis* subtypes and IBS pathogenesis.

In the current study we have identified *Blastocystis* subtypes using a fixed commercial primer kit (ST1–ST7) to cover the most prevalent subtypes among the human host, particularly ST3. It must be acknowledged, however, that rare subtypes and mixed infections cannot be identified using the applied kit, but rather by sequencing, which remains the ultimate reference standard for subtyping. Another limitation that must be stated is the relatively small number of patients infected with ST1 which limits the statistical power of subtype-specific comparisons. Since patient recruitment was performed prospectively without prior knowledge of parasite subtype, ST3 and ST1 group sizes varied considerably reflecting the higher prevalence of ST3 as reported in several studies. Therefore, all subtype-associated findings including clinical observations and IL-8 levels serve as exploratory findings rather than definitive causal associations. It is also to be noted that the results of the semi-quantitative assessment of reducing sugar and stool pH should be cautiously described as broad metabolic observations rather than definite mechanistic indicators. Additionally, the absence of microbiome analysis and dietary assessment further constrains the interpretation of metabolic and inflammatory findings. Future studies incorporating longitudinal designs, larger multicenter cohorts, and integrated microbiome and immunological profiling are warranted to better elucidate the role of *Blastocystis* in IBS pathogenesis.

## Data Availability

The data supporting the findings of this study are available from the corresponding author upon reasonable request.
